# The psychometric properties of the Arabic version of the Teachers’ Attitudes towards Differentiated Instructional Scale

**DOI:** 10.3389/fpsyg.2025.1425152

**Published:** 2025-08-22

**Authors:** G. H. Alnahdi, Marcela Pozas, Mona F. Sulaimani, V. Letzel-Alt

**Affiliations:** ^1^Department of Special Education, College of Education, Prince Sattam Bin Abdulaziz University, Al-Kharj, Saudi Arabia; ^2^Department of Education and Social Work, Faculty of Humanities, Education and Social Sciences, Institute for Teaching and Learning, University of Luxembourg, Luxembourg, Luxembourg; ^3^School of Psychology, University of Monterrey, Monterrey, Mexico; ^4^Department of Special Education, Faculty of Education, King Abdulaziz University, Jeddah, Saudi Arabia; ^5^Institute of Educational Sciences, Leuphana University Lüneburg, Lüneburg, Germany

**Keywords:** TAT-DIS, differentiated instruction, teachers’ attitudes, psychometric properties, inclusive education

## Abstract

According to literature, teachers’ attitudes are a strong predictor of their differentiated instructional practice. However, empirical research exploring teachers’ attitudes, specifically towards the practice of DI, is still quite limited. Currently, there is only one instrument available that assesses teachers’ attitudes towards DI, that is the Teachers’ Attitudes towards Differentiated Instructional Scale (TAT-DIS), which has not been explored within any Arabian country. With this background, this study examines the psychometric characteristics of the Arabic version of the tool. A total 221 teachers in two Arabic countries, Egypt and Saudi Arabia, participated in the study. Results of the confirmatory analysis confirmed the hypothesized two-factor structure and internal consistencies of the subscales were good. Limitations and implications of the study are further discussed.

## 1 Introduction

In order to meaningfully support students’ broad array of learning needs, teachers are urged to differentiate their instruction in their daily teaching practice. Teachers are “the predominant actors in setting the nature of the classroom environment” (OECD, 2023, p. 187), and therefore play a pivotal role when it comes to the teaching practice of differentiated instruction (DI). With this background, there has been a substantial output in research exploring the associations between teacher-related variables that have an impact in their DI practice (Letzel, 2021), such as their preparedness and theoretical knowledge of DI (Pozas and Letzel, 2020; Wan, 2017), beliefs (Wan, 2016; Whitley et al., 2019), resources (Schwab et al., 2020), readiness (Adams et al., 2021) and self-efficacy (Dixon et al., 2014; Knauder and Koschmieder, 2019; Suprayogi et al., 2017). On the other hand, previous research has also discussed the predictive role of teachers’ attitudes on their DI practice (de Boer et al., 2011; Letzel et al., 2022; Plunkett and Kronborg, 2019; Rutigliano and Quarshie, 2021; Schwab et al., 2019; Whitley et al., 2019).

Given the important role that teachers’ attitudes have on their DI practice, and considering that DI is a key instructional approach that ensures inclusive education (OECD, 2023), it is important to be able to measure teachers’ attitudes towards DI. This is return allows to identify any barriers hindering the successful implementation of DI and inclusive education policies can be identified and addressed (Ewing et al., 2018). In this vein, appropriate tools are needed that allow measuring these domain-specific attitudes in teachers as well as can be used for international comparative analyses in order to “provide insight into factors that shape participants’ attitudes” (Sharma et al., 2018) in different cultural contexts as well as identify and implement interventions aimed developing the future teacher force. The Teachers’ Attitudes towards Differentiated Instructional Scale (TAT-DIS) is a recently developed, and currently, the only available measurement tool that aims at exploring teachers’ attitudes towards DI (Letzel et al., 2020). Hence, the present study aimed to explore the psychometric properties of the TAT-DIS within an Arabic teacher sample in Egypt and Saudi Arabia, which are amongst the largest Arab countries. Although differences across both countries’ educational systems are to be expected, these are relatively small. Egypt and Saudi Arabia share a common language and religious context, their educational systems have both similarities and differences. Historically, a substantial number of Egyptian educators have worked in Saudi Arabia, particularly over the past five decades. Many teacher preparation programs and instructional practices in Saudi Arabia were influenced by Egyptian academics and practitioners. This shared professional foundation has contributed to considerable overlap in pedagogical approaches. However, differences remain—particularly in terms of infrastructure, teaching materials, and technological resources—where Saudi Arabia has made significant advancements in recent years. Although some scholars, such as BouJaoude and Gholam (2014), have suggested that teaching and learning processes in both Egypt and Saudi Arabia incorporate cooperative learning and hands-on approaches, the reality in many classrooms remains markedly different. Instruction in both countries continues to be largely dominated by teacher-centered methods, with passive student engagement and traditional lecturing still prevailing as the primary mode of delivery.

### 1.1 Differentiated instruction

DI is pedagogical inclusive approach that recognizes and addresses student diversity by effectively adapting the learning environment, instructional methods, tasks and materials according to each student’s learning needs (Valiandes et al., 2018). In this line, DI strives to establish educational equity for students by fostering learning at their own rate, and thus, as efficiently as possible (Gheyssens et al., 2023). As a result, researchers and policymakers consider DI as key teaching quality domain (Maulana et al., 2020), and thus, has been in different international teaching quality model conceptualizations (Bell et al., 2019; Praetorius et al., 2018; Van de Grift et al., 2014). Results from a cross-country study by Maulana et al. (2020) showed that both South Korean and Dutch teachers consider DI as a key feature for effective teaching. However, Maulana’s et al. (2023) recent comparison study across five countries indicates that there is some degree of variations when it comes to teachers DI implementation. Thus, it can be concluded that although DI is acknowledged as an international criterion of effective teaching, its implementation varies significantly across countries and depends on teachers interpersonal and intrapersonal characteristics.

There is a variety of scholarly literature that suggest numerous ways in which educators can differentiate instruction, for example, through the use of tiered assignments, flexible grouping, mastery learning, staggered nonverbal learning aids, peer tutoring and open education strategies such as station learning (Lawrence-Brown, 2004). Although there are various ways as to how teachers can differentiate instruction, selecting and implementing the best practice will strongly depend on the students’ needs as well as the classroom’s resources and constraints. Consequently, DI requires for teachers to conduct continuous monitoring and assessing of their students’ progress (Keuning and Van Geel, 2021) through formative assessment in order prepare their lessons and identify whether a student requires further support. Taken together, for teachers to meaningfully accommodate to their students’ broad array of learning needs, they require a highly developed diagnostic competence. However, teachers struggle with the regular diagnostic assessment of students’ individual learning needs as well as development (Gaitas and Alves Martins, 2017) and commonly rely other unsystematic evaluations (Smets and Struyven, 2020).

Empirical findings stemming from international research have revealed that DI is positively associated with students’ achievement (Goddard et al., 2015; Bal, 2016; Valiandes, 2015). For instance, Puzio’s et al. (2020) systematic review and meta-analysis revealed that DI is an effective practice as it fostered primary school students’ literacy outcomes. Furthermore, research has also shown a positive impact of DI on students’ non-achievement outcomes such as school well-being and academic self-concept (DeVries et al., 2018; Pozas et al., 2021; Venetz et al., 2019) as well as their mathematics self-efficacy (Lai et al., 2020). In the particular case of Arabian countries, studies have also shown that DI has benefits for students’ learning. For instance, a recent study by Al-Shehri et al. (2020) revealed that Saudi Arabian sixth grade science students had an improvement in their level of critical thinking skills after a differentiated instructional program. Likewise, findings from Magableh and Abdullah (2020) showed that eight grade students in Jordan had a significant development in their English language skills when being taught in a differentiated classroom. Nevertheless, Deunk et al. (2018) have also reported that teachers’ DI does not always lead to positive achievement and non-achievement student outcomes. For example, studies by DeVries et al. (2020) and Gehrer and Nusser (2020) revealed that students did not benefit, in general, from DI in the subjects of Math and German. In light of these heterogeneous results, Kupers et al. (2023) argue that DI should be conscientiously planned and in line with other key domains of effective teaching, such as classroom management and cognitively activating tasks (Holzberger et al., 2019). However, despite its recognized benefits, implementing DI in Arab countries might pose challenges related to the large class sizes, limited teacher training in inclusive practices, and insufficient classroom resources (Schwab et al., 2020). For instance, Alnahdi et al. (2024) reported that teachers in Saudi Arabia often perceive a lack of practical tools and administrative support for DI. Moreover, international findings have shown that DI might not always yield consistent positive outcomes.

## 2 Teacher attitudes and DI

As described above, the implementation of inclusive teaching practices strongly depends on the teachers’ attitudes. However, it is of upmost important to highlight that all of these abovementioned studies have used instruments that aim to assess teachers’ attitudes towards inclusion in general (e.g., Seifried, 2015), attitudes towards student heterogeneity (Gebauer et al., 2013) or attitudes towards inclusive education (for an overview please see Kopmann and Zeinz, 2016) and not to the specific domain of DI. With this context, it is important for professionals and researchers to have assessment tools that specifically focus on the different sub-dimensions of teachers’ attitudes (Savolainen et al., 2022) such as the attitude object of DI. Using reliable scales to measure teachers’ attitudes towards DI is an essential step in understanding teachers’ perceptions and experiences when differentiating their instruction. Given the need for an appropriate and reliable instrument, Letzel et al. (2020) set out to develop and validate an instrument aimed at specifically assessing teachers’ attitudes towards their practice of DI. The TAT-DIS is a simple, reliable and easily administrable questionnaire consisting of eight items and designed to measure the domains of teachers’ value of DI and perceived insufficient resources. This scale has already been used in different German teacher samples (Letzel et al., 2022; Pozas et al., 2022) and has been recently adapted and validated in a Chinese teacher sample Bi et al. (2024). A significant result from Letzel’s et al. (2022) study is the fact that teachers identify both the “positive” and the “negative” aspect of DI, and more importantly, they can recognize both attitude domains (value of DI and perceived insufficient resources) towards DI in a similar or different level. Such findings underline the theoretical considerations by Wilson et al. (2000) which established that “people can simultaneously hold two different attitudes toward a given object in the same context” (Ajzen, 2001, p. 29). Additionally, Letzel’s et al. (2022) study revealed that, teachers’ who score higher in the subscale of value of DI, tend to differentiate their instruction more often. Similar results were also found in the study by Pozas et al. (2022). In Bi’s et al. (2024) study, results showed that Chinese teachers hold high levels of value of DI as well as perceived insufficient resources. Furthermore, the authors also indicate that in comparison to secondary school Chinese teachers, primary school Chinese teachers hold higher levels of both value of DI and perceived insufficient resources.

## 3 Purpose and research question

In order to support the learning and well-being of all learners, teachers are required to implement DI (OECD, 2023). As numerous studies have shown, teachers’ attitudes play a vital role in their in-class instruction, such as the inclusive teaching practice of DI. However, while questionnaires measuring teachers’ attitudes towards inclusion as well as inclusive education and student diversity exist, up to now, there is only one instrument measuring teachers’ attitudes specifically towards the practice of DI, which is the Teachers’ Attitudes towards Differentiated Instructional Scale (TAT-DIS) (Letzel et al., 2020). The TAT-DIS has been originally published in German and English, and has been recently adapted and validated within the Chinese mainland context (Bi et al., 2024). Considering as well that teacher attitudes significantly differ “across countries, cultures and educational systems” (Saloviita, 2020, p. 64), having validated versions of the TAT-DIS for cross-cultural research purposes are of relevance. Accordingly, adapting TAT-DIS into an Arabic version and evaluating it seems worthwhile. In this sense, the present study is guided by the following research question: Does the Arabic version of the TAT-DIS pre-serve the psychometric properties of the original version of the scale?

## 4 Methods and materials

### 4.1 Participants and procedure

The sample of this study consists of 221 teachers (Table 1) from Egypt (120) (30 males and 90 females) and 101 from Saudi Arabia (22 males and 79 females). A total of 75% of the teachers hold a bachelor’s degree while the rest of the participants hold another type of higher education degree (ranging from a high diploma to a doctoral degree). Data collection was conducted during the summer and fall of 2022. Teachers from both countries were invited to fill out a voluntary online survey, which took approximately 15 to 20 min.

**Table 1:** Descriptive statistics of participants by country, gender, and teaching level.

Country	Male	Female	Primary school	Secondary school
Egypt (*n* = 120)	30	90	70	50
Saudi Arabia (*n* = 101)	22	79	60	41

### 4.2 Instrument: Teachers’ Attitudes towards Differentiated Instructional Scale

The TAT-DIS consists of eight items grouped into two subscales: value of DI (VDI; five items) and perceived insufficient resources (PIR; three items) (Letzel et al., 2020). The items’ response format is based on five response levels, ranging from 1 = “strongly disagree” to 5 = “strongly agree.” One of the items on the value of DI sub-scale uses reverse scoring, thus its score has to be converted before the analysis. In this sense, high scores on the value of DI scale are an indicator of positive attitudes towards the practice of DI, whereas high scores on the perceived insufficient resources sub-scale indicate high negative attitudes.

#### 4.2.1 Translation of the TAT-DIS

Following a back-translation approach, the scale’s translation was executed in multiple steps (see Beaton et al., 2000). In an initial step, three bilingual translators translated the items from English to Arabic. Afterwards, the resulting different Arabic versions were combined into one. Next, two other bilingual academics back-translated the Arabic version into English and the resulting two translated English versions were combined into one and then compared to the original English version. After ensuring that the back-translation kept the same meaning for all items, a pilot sample (*n* = 66) was used to check the clarity of all items and to revise for internal consistency (*α*VDI = 0.96 and *α*PIR = 0.91). Two of the original authors of the TAT-DIS provided support and assistance throughout the process.

Although standard Arabic was used in the translation to ensure cross-country comprehensibility, care was taken to choose terminology that is widely understood and neutral across regional dialects in Egypt and Saudi Arabia. For instance, we selected the term *‘التدريس المتمايز’* (differentiated instruction) consistently across items, as it conveys the intended pedagogical concept without ambiguity, unlike regional expressions such as “*تفريق الشرح*” or *“تنويع التعليم*”, which may hold different or unclear meanings in one context or another. Similarly, verbs such as “*أفرق في طريقة تدريسي*” and “*إعداد خطط دروس متباينة*” were chosen for clarity and formality, avoiding dialectal phrasing that could reduce conceptual precision. A pilot test with teachers from both countries confirmed that all items were clearly understood and appropriate within their educational and linguistic contexts.

## 5 Results

To answer the study’s research question, confirmatory factor analyses (CFA) were conducted using Amos 21 software. Tests of reliability and other descriptive statistics were conducted using SPSS 21.

### 5.1 Confirmatory factor analysis and reliability

In order to evaluate the psychometric properties of the TAT-DIS’ Arabic version, structural and convergent validity were examined through a confirmatory factor analysis (CFA) (de Vet et al., 2011). Following the proposed model by Letzel et al. (2020), a two-factor-correlated model was tested. To evaluate whether the observed data of this study would fit the hypothesized model, different fit indices were examined for: (a) a ratio ≤3 for *χ*^2^/df ratio, (Byrne, 2004), (b) and value ≤0.08 for root mean square error of approximation (RMSEA) and for standardized root mean square residual (SRMR) (Hu and Bentler, 1999) and (c) values of ≥0.9 for comparative fit index (CFI) and Tucker–Lewis index (TLI) (Hu and Bentler, 1999).

As seen from Table 2, except for the SRMR, the fit indices for the two-factorial model indicated a good model fit (*χ*^2^ = 57.048, df = 19, *p* ≤ 0.001; RMSEA = 0.09, CFI = 0.93, TLI = 0.93, SRMR = 0.73). In particular, the resulting CFI for the two-subscale model with all eight items can be considered as a good indicator that the observed data did indeed fit the hypothesized model. Thus, it can be concluded that the fit is acceptable. Furthermore, as indicated by the Cronbach’s *α*, the two factor’s internal consistencies were at an acceptable level (value of DI *α* = 0.85; perceived insufficient resources *α* = 0.87). In addition, the composite reliability for the subscale of value of DI was 0.79 and the average variance extracted was 0.48. For the subscale of perceived insufficient resources, the composite reliability was 0.60 while the average variance extracted was 0.381.

**Table 2:** CFA model of TAT-DIS-AR.

Model	*χ*^2^	df	*p*	CFI	TLI	SRMR	RMSEA	90% CI for RMSEA	
								LL	UL
Eight items	57.048	19	0.001	0.93	0.93	0.73	0.09	0.068	0.124
Eight items^a^	50.885	18	0.001	0.94	0.91	0.73	0.09	0.062	0.121
Six items	23.814	8	0.002	0.97	0.94	0.38	0.09	0.052	0.140
Six items^b^	15.447	7	0.031	0.98	0.97	0.34	0.07	0.021	0.124

CFI, comparative fit index; TLI, Tucker–Lewis index; SRMR, standardized root mean square residual; RMSEA, root mean square error of approximation; CI, confidence interval; LL, lower limit; UL, upper limit.

a Error from items within each subscale were covariate to improve the fit.

b Error were covariate for two items within the same domain.

**Figure 1:**
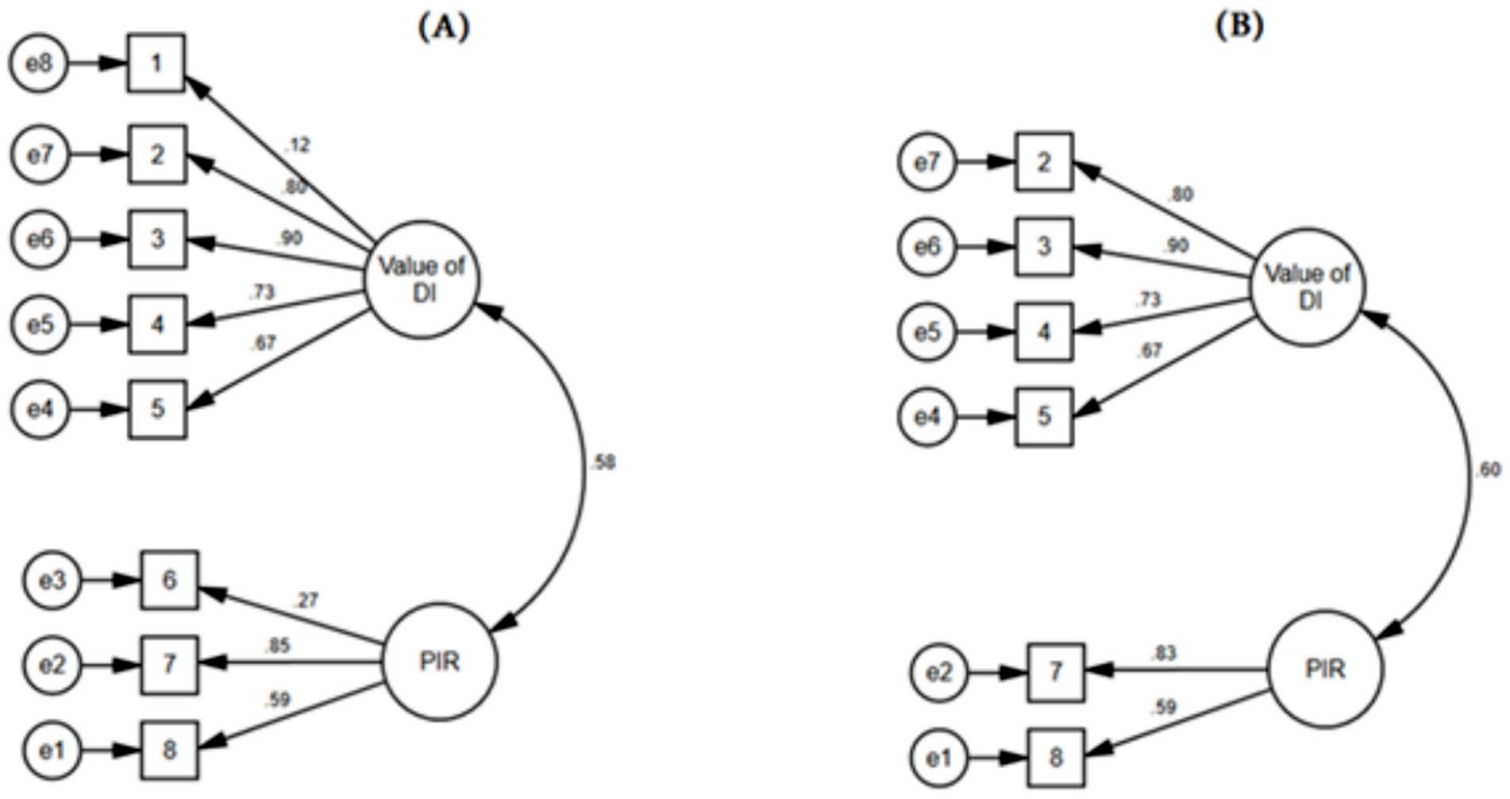
Confirmatory factor analysis for two models. **(A)** Includes all eight items. **(B)** Excludes two items.

However, important to note is in each of the subscales, there was an item that had very low factor loading. As observed in Figure 1, these are item 1 corresponding to the subscale of value of DI “I do not see a reason why I should differentiate my instruction” (0.12) and item 6 for the subscale of perceived insufficient resources “I do not have enough time to differentiate my instruction as I often as I want to” (0.27). Taking this into consideration, a second model was calculated with just six items. Although the goodness of fit decreased for the two-factor model with 6, the fit indices still indicate an acceptable fit (*χ*^2^ = 23.814, df = 8, *p* ≤ 0.01; RMSEA = 0.09, CFI = 0.97, TLI = 0.94, SRMR = 0.38). In addition, the composite reliability for the value of DI subscale was 0.86 and the average variance extracted was 0.61 (>0.5). In sum, it can be concluded that the six-item scale showed as well positive indicators for convergent validity and reliability (Hair et al., 2010).

### 5.2 Descriptive statistics

Table 3 shows the means and standard deviations of the TAT-DIS-AR subscales. The two TAT-DIS-AR means were high and with homogeneous standard deviations. As the theoretical mean of the subscales was 3, the scores were significantly positive: value of DI [*t*(672) = 24.66, *p* ≤ 0.001, *d* = 1.54] and perceived insufficient resources [*t*(215) = 17.18, *p* ≤ 0.001, *d* = 1.16].

**Table 3:** Descriptive statistics by gender and whether teaching SEN students.

	Value of DI	Insufficient resources
Valid	219	216
Mean	3.94	3.76
Std. deviation	0.61	0.65

Gender				
	Male	Female	Male	Female
Valid	52	167	52	164
Mean	3.90	3.95	3.75	3.76
Std. deviation	0.43	0.65	0.61	0.67

Working with SEN students				
	Yes	No	Yes	No
Valid	57	121,954	56	153
Mean	3.96	3.94	3.49	3.85
Std. deviation	0.55	0.61	0.78	0.58

To sum up, these results indicate that the majority of the participating teachers in this sample have a high value of DI, but also a high level of perceived insufficient resources.

## 6 Discussion

Teachers’ domain-specific attitudes are a key predictor for their teaching behavior, such as the practice of DI. Thus, in order to explore and be able to develop comparative analyses, a reliable and valid measurement tool is required. With this background, the present study examined the factorial structure and reliability of the TAT-DIS-AR using a sample of teachers from two different Arabic regions.

The hypothesized two-factor of the TAT-DIS was confirmed by the results of the CFA for the TAT-DIS-AR (Letzel et al., 2020). However, although the TAT-DIS-AR CFA model fit indices indicated an acceptable fit, two items (see Figure 1) showed to have a low loading in each of the two subscales. As mentioned in the previous sections, the TAT-DIS has been already explored within a Chinese sample. Results from Bi’s et al. (2024) study replicated the findings from the original German study (Letzel et al., 2020) and all eight items’ factor loadings were higher than 0.30 which indicates an excellent loading across their target factor (Pallant, 2020). Previous international research has shown that teachers’ attitudes, and in particular within the field of inclusive education, are the result of a complex interplay of demographic variables but also cultural background (Cullen et al., 2010; Leyser et al., 2011; Savolainen et al., 2012; van Steen and Wilson, 2020). Thus, it can be assumed that such low item loadings could be deriving from specific cultural factors. Another possible explanation is that both of these items are negatively phrased. As seen from previous studies exploring the psychometric properties of instruments in from different fields and context (Alnahdi, 2019, 2020, 2024), negatively worded items could result in having “artifactual factor” (Spector et al., 1997). In this context, although the results support the structural validity of TAT-DIS-AR, it is necessary that further research within other Arabic teacher samples continues to explore whether items 1 and 6 still have modest loading on the corresponding subscales. Until that happens, it is strongly recommended that researchers using the TAT-DIS-AR employ the scale without these two items for purposes of exploring the Arabic context solely as it shows a better-fit index. On the other hand, in case that researchers aim to have comparable data that will be used for cross-country comparison analyses [e.g., China Bi et al. (2024)], it is suggested to employ the complete eight item scale as the fit indices as a whole were good.

In addition to the factorial structure, the reliability of the TAT-DIS-AR was examined. In the present sample, the reliability analyses of the two subscales indicated high internal consistencies. Similar to the studies by Letzel et al. (2020) and Bi et al. (2024), the Cronbach’s alpha of the subscales value of DI and perceived insufficient resources were above 0.70.

Interpreting the descriptive scores, it can be concluded that while Egyptian and Saudi Arabian teachers see the value of implementing DI, they also perceive insufficient resources when differentiating their instruction. These results are consistent with findings from Letzel et al. (2020, 2022) in Germany and Bi et al. (2024). In addition, such results also support Letzel’s et al. (2022) discussion that “distinguishing just between positive or negative attitudes might not shed sufficient information on the relationship between teachers’ attitudes and their DI use” (p. 10). In this context, it can be assumed that teachers are able to distinguish between the positive and negative aspects of DI. However, further research is needed in order to examine in detail the manifestations of attitudes, their development and their impact on their teacher behavior. Additionally, as previous studies developed in other countries have shed similar results, the present study also calls for further cross-country comparisons with regards to teachers’ attitudes towards DI.

To finalize, it is necessary to mention that the present study underlines several limitations. First, the study’s sample consists of two countries of the Arabic world. Thus, further studies exploring other Arabic samples are needed to further confirm the present study’s findings. Second, the present study did not conduct further measurement invariance across demographic variables such as gender nor amongst teachers’ educational stages (i.e., primary and secondary school). Given that strong or scalar measurement invariance is a prerequisite for calculating meaningful mean comparisons across groups, it is important that further studies conduct such analyses. Third, the results were based solely on cross-sectional analysis. Therefore, inferences about teachers’ attitudes towards DI have to be done with caution. In this sense, research should aim to follow to follow a longitudinal design to investigate the development of the TAT-DIS dimensions. Lastly, given the results from the present study, the present study calls for more replication studies using the TAT-DIS-AR version. Furthermore, it is strongly suggested for measurement invariance analyses to be conducted across the sample (e.g., gender, educational stages, and countries) in order to be able to further explore potential differences within the sample.

## 7 Conclusion

In conclusion, this study found that the TAT-DIS-AR is a reliable instrument for the assessment of teachers’ attitudes with regards to the inclusive practice of differentiated instruction in their classrooms. Therefore, this scale can be recommended for use in future studies. This provides the opportunity not only to contributes to building a more detailed understanding of teachers’ attitudes and perceptions regarding DI, but also to conduct future international comparison research that could help generate a more comprehensive understanding on how DI is understood, experienced and practiced around the world.

## Data Availability

The raw data supporting the conclusions of this article will be made available by the authors, without undue reservation.
